# Supplementary material for: Demand prediction for shared bicycles around metro stations incorporating STAGCN
Source: PLoS One. 2025 Jul 15;20(7):e0328452. doi: 10.1371/journal.pone.0328452 (PMC12262888; doi:10.1371/journal.pone.0328452)
Supplement: Attachment — Submitted filename: Response to Reviewers.pdf [file pone.0328452.s003.pdf]

Response to Reviewers for Manuscript ID: PONE-D-25-16186

Dear Editors and Reviewers,

We would like to sincerely thank you for your time and effort in reviewing our manuscript titled "Demand Prediction for Shared Bicycles around Metro Stations Incorporating STAGCN". We greatly appreciate your insightful comments, which have significantly helped us improve the quality of our paper. We have addressed all the points raised by the reviewers and have made the necessary revisions accordingly.

Journal Requirements:

1.Please ensure that your manuscript meets PLOS ONE's style requirements, including those for file naming.

Response:We have verified that the manuscript meets the requirements.

2.Please note that PLOS ONE has specific guidelines on code sharing for submissions in which author-generated code underpins the findings in the manuscript. In these cases, we expect all author-generated code to be made available without restrictions upon publication of the work.

Response:The project code has been packaged and uploaded to the support information file.

3.Thank you for stating the following financial disclosure: [Science and Technology Development Plan Project of Jilin Province (20210101416JC Education Industrial Cultivation Project of Jilin Province (JJKH20230306CY)].

Please state what role the funders took in the study. If the funders had no role, please state: ""The funders had no role in study design, data collection and analysis, decision to publish, or preparation of the manuscript.""

If this statement is not correct you must amend it as needed.

Please include this amended Role of Funder statement in your cover letter; we will change the online submission form on your behalf.

Response:We have added the following explanation in the cover letter:"The funders had no role in study design, data collection and analysis, decision to publish, or preparation of the manuscript."

4.Thank you for uploading your study's underlying data set. Unfortunately, the repository you have noted in your Data Availability statement does not qualify as an acceptable data repository according to PLOS's standards.

At this time, please upload the minimal data set necessary to replicate your study's findings to a stable, public repository (such as figshare or Dryad) and provide us with the relevant URLs, DOIs, or accession numbers that may be used to access these data.

Response: The dataset required for the results of this study has been uploaded to a stable , publicly accessible repository. The website address is
https://doi.org/10.34740/kaggle/dsv/12046915

Comments to the Author

Reviewer #1: The manuscript is technically clear, sound and well written. The data is available

online without any restriction. I would like to recommend you to add the graphs of your comparative results along with the tables.

Response: We are very pleased that you think our manuscript is clear, reliable and well-written in terms of technology. Thank you for your valuable feedback. We have made corresponding revisions to the manuscript based on your suggestions. The added charts include Fig 7, Fig 9, Fig 11 and Table 2, Table 5, Table 6.

Reviewer #2: The manuscript addresses an important problem by focusing on demand forecasting for shared bicycles around metro stations to improve metro connectivity. The identification of metro-connected trips and the integration of STAGCN, LSTM, and Informer models are valuable technical elements.

However, several areas require improvement:

Thank you to the reviewer for your meticulous review and valuable suggestions of our paper. Your opinions are of great significance in helping us improve the paper. Below are our specific responses and explanations for the suggestions you raised:

1.Literature Review Structure and Analytical Depth:

The literature review mainly describes what existing studies have done but lacks an analytical perspective on research gaps. I recommend restructuring it with a literature review table summarizing prior work's methods, assumptions, and focus areas, and clearly identifying the gaps. This would better position the specific contribution of integrating metro connectivity within the broader context.

Response: We fully agree with this suggestion and have made significant revisions to the Related work (Section 2). The specific measures include:

In the summary of each study, an analysis of the advantages and disadvantages of the methodology has been added.

The literature review is divided into two themes: research on bike-sharing metro connectivity trip identification, research on demand prediction methods for shared bikes. Under each theme, key methods, assumptions, and limitations have been summarized.

A new section titled "Research Gaps" (Section 2.3) has been added, clearly indicating the shortcomings of existing research in terms of the accuracy of travel identification for the connection, complex spatio-temporal modeling, and efficiency of long sequence prediction, and explaining how this study fills these gaps.

2.Clarifying the Contribution:

Currently, the contribution is stated more as a description of the work completed rather than highlighting the true research contribution. The paper should explicitly articulate what gap it addresses and how it advances knowledge compared to existing studies on shared bike demand forecasting.

Response: We have, in accordance with your suggestions, strengthened the description of the contributions in the contribution statement, clearly stating the research question (the accuracy issue of subway connection with bike demand prediction) and the shortcomings of the existing bike demand prediction methods (insufficient spatio-temporal dependency modeling), and clearly elaborating on the uniqueness and knowledge advancement of this paper's contributions.

3.Managerial Insights and Practical Framework:

Providing managerial insights on how operators can use the proposed model, along with a conceptual framework for practical deployment, would strengthen the real-world relevance.

Response: Based on your advice, We further optimized the content in the summary section of the paper, elaborating in detail on how precisely predicting the short-distance bike-sharing demand in the vicinity of subway stations can help bike-sharing companies optimize their vehicle scheduling strategies, reduce vehicle shortages or idleness during peak hours, thereby improving user efficiency and enhancing urban order management. Additionally, we also explored the integration of demand prediction technology with smart city systems, proposing to dynamically adjust vehicle placement positions through real-time passenger data and electronic fence technology, in order to reduce operating costs and improve resource utilization, and promote the industry's transformation towards refined and intelligent operations.

4.Visualization and Result Analysis:

Result presentation should be improved, both visually and analytically. Beyond graphical comparisons, analyzing key factors influencing prediction accuracy would enhance understanding.

Response: Following the reviewers' suggestions, we have made improvements to the presentation of the results. The specific measures are as follows:

Enhanced visualization: we added Fig 9, which presents the correlation between time period, temperature, weekend and the demand for shared bikes in an intuitive way, enabling a clearer depiction of the relationships between the variables.

Deepened analysis: we further analyzed the impact of time period, temperature, weekends, etc. on the demand for shared bikes, and explored the underlying mechanisms behind these factors. For instance, changes in temperature may be related to external activities (such as outdoor sports and outings), while the morning and evening rush hours are closely associated with commuting needs.

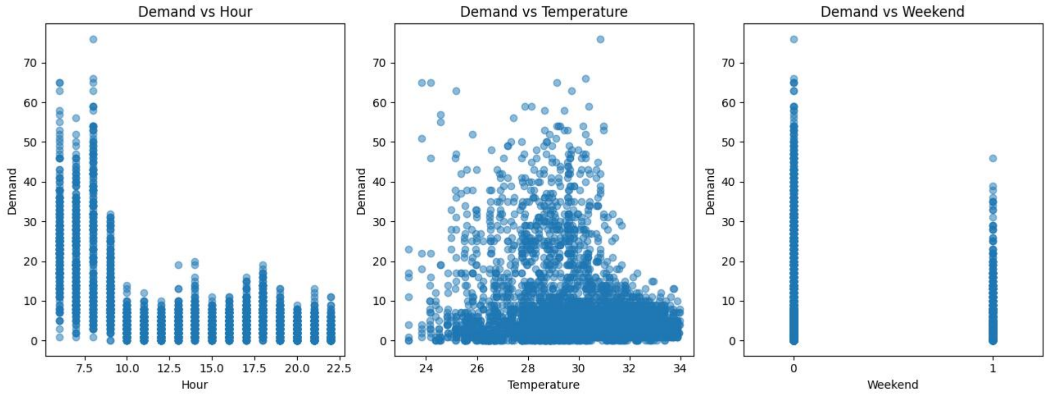

Fig 9. Correlation feature scatter plot

5.Relevant Studies:

The following papers may help enrich the analysis:

Ma, X., Yin, Y., Jin, Y., He, M., & Zhu, M. (2022). Short-term prediction of bike-sharing demand using multi-source data: a spatial-temporal graph attentional LSTM approach. Applied Sciences, 12(3), 1161.

Yu, L., Feng, T., Li, T., & Cheng, L. (2023). Demand prediction and optimal allocation of shared

bikes around urban rail transit stations. Urban Rail Transit, 9(1), 57-71.
Hu, J., Wagner, F., Ayargarnchanakul, E., Nachtigall, F., Milojevic-Dupont, N., Lu, C., & Creutzig, F. Examining Urban Form Effects on Integrated Use Demand of Bike-Sharing and Metro. Available at SSRN 4985690.
Zi, W., Xiong, W., Chen, H., & Chen, L. (2021). TAGCN: Station-level demand prediction for bike-sharing system via a temporal attention graph convolution network. Information Sciences, 561, 274-285.

Response: Thank you very much for the relevant literature you provided. After reading and analyzing these documents, we found that they indeed offered useful perspectives and supplements to certain aspects of this research. Especially in the research on the demand prediction of shared bikes. Based on your suggestions, we have appropriately cited these documents in the literature review (such as [16], [21], [34], [35]).

Reviewer #3: This manuscript addresses the important challenge of accurately forecasting dockless bike-sharing demand around metro stations. The proposed methodology is structured as a three-stage pipeline: (1) a KDTree-based approach to identify actual metro-bicycle transfers and determine an optimal buffer radius; (2) a deep hybrid model incorporating Spatio-Temporal Attention Graph Convolutional Networks (STAGCN), LSTM, and Informer components; and (3) empirical evaluation using a two-month dataset from Shenzhen. While the integration of graph learning, attention mechanisms, and long-sequence forecasting is promising, several aspects require clarification, justification, and methodological enhancement.

The authors are grateful for the time and effort that the reviewer dedicated to lyingand improving this article. Thank you for your comments, we corrected our manuscript accordingly.

Major comments:

1.While KDTree is applied to improve over fixed-radius methods, the final model still employs a uniform 300-meter buffer. The added value of KDTree is not fully demonstrated. Why not explore learning flexible, irregular catchment zones from the data? At minimum, comparing the optimal radius found to alternatives like polygonal catchment boundaries could enhance the rigor.

Response: In response to the comments raised by the reviewers, we have made the following revisions:

To enhance the rigor of the identification method in this paper, we have added a comparative experiment. We compared the Baseline (300-meter buffer zone), the Voronoi polygon, and the improved method in this paper. The results in Table 1 show that, compared with the Baseline (300-meter buffer zone) method, this paper has eliminated approximately 24% of non-connecting trips. Compared with the Voronoi polygon, this method proposes approximately 26% of non-connecting trips, effectively reducing non-connecting trips within fixed buffers and irregular polygons.

The main role of the KDTree algorithm in this study is to perform neighbor matching. Through this algorithm, we can efficiently match the nearest subway station for the starting and ending points of each shared bicycle order, and calculate the Euclidean distance between the starting point and the ending point and the subway station, thereby screening out the eligible transfer travel orders that meet the conditions.

The optimal transfer buffer radius adopted in this study is dynamically determined through an

analysis method based on the order growth volume. Specifically, we use ArcGIS software to build multi-ring buffers around the subway stations and conduct intersection analysis on each radius range of shared bicycle orders, and count the growth pattern of the order volume. When the growth rate of orders in a certain radius range begins to slow down, it indicates that the distance corresponding to this radius is the optimal transfer radius for subway travel. As shown in Fig 6 and Fig 7, we concluded that 300 meters is the optimal transfer buffer radius.

Table 1. Identification result of connecting shared bike to metro

date	Baseline (300 m buffer)	Voronoi	Proposed	Efficiency ratio
2021/5/6	84567	87923	64855	76.69%(Baseline)，73.76%(Voronoi)
2021/5/7	85382	89456	65386	76.58%(Baseline)，73.09%(Voronoi)
2021/5/8	84587	86581	65073	76.93%(Baseline)，75.16%(Voronoi)
2021/5/9	66575	68253	50504	75.86%(Baseline)，73.99%(Voronoi)
2021/5/10	86768	88776	64998	74.91%(Baseline)，73.21%(Voronoi)
total	407879	420989	310816	76.21%(Baseline)，73.82%(Voronoi)

2.The identification of the inflection point on the order-growth curve lacks a formal definition. The method appears heuristic. A sensitivity analysis showing how prediction accuracy varies with small radius changes would strengthen confidence in the selected threshold.

Response: We sincerely thank the reviewers for their valuable suggestions. We have carefully revised according to your comments, and the specific improvements are as follows:

Inflection point formal definition: We calculated the rate of change (slope) of the total order volume relative to the buffer radius using the numerical differentiation method. We found that the slope significantly decreased around 300 meters, indicating that the order growth rate was stabilizing.

Sensitivity analysis: To verify the robustness of the 300-meter radius, we identified the pick-up order volume at intervals of 10 meters within the range of 250 meters to 350 meters, and calculated the percentage change relative to the order volume at 300 meters. The results showed that the order volume changed slightly around 300 meters, confirming the stability of this radius.

Chart improvement: Add Fig 7: (a) shows the increase in total outbound orders and their rate of change, highlighting the slowdown in the growth rate at 300 meters; (b) presents the sensitivity analysis around 300 meters, showing the percentage change in order volume with minor changes in the radius.

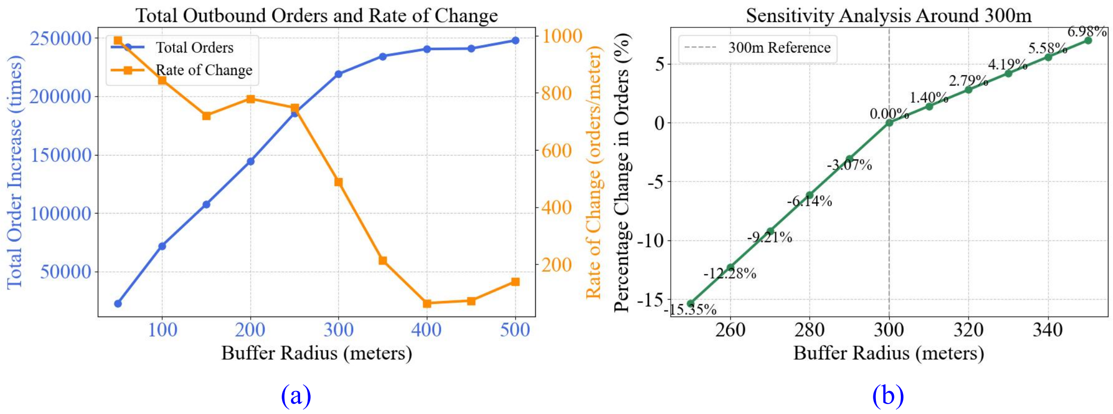

Fig 7. (a) Order growth volume and growth rate, (b) Sensitivity analysis near 300 meters

3.The paper does not clearly describe how the spatial adjacency matrix is computed. Is it based on physical metro-line connections, Euclidean distance, demand correlation, or another metric? Explicitly detailing this step is critical for reproducibility and understanding the spatial relationships modeled.

Response: We would like to express our gratitude to the reviewers for their valuable suggestions. Regarding the calculation of the spatial adjacency matrix, we employed a method based on the Euclidean distances between subway stations and the network structure of subway line connections. Specifically, the elements of the adjacency matrix are derived by calculating the Euclidean distances between stations and considering the connectivity of subway lines. If the Euclidean distance between two stations is less than a preset threshold and there is a direct subway line connection between them, the corresponding element value in the adjacency matrix is 1, indicating a spatial dependency relationship between them. This construction method effectively captures the spatial connections between stations and supports subsequent graph convolution operations.

4.While time-of-day, temperature, and weekend indicators are selected based on Pearson correlation (|ρ|>0.15), the exclusion of other weather variables like rainfall and humidity is not justified. Were these omitted due to multicollinearity, missing data, or weak correlations?

Response: In response to your suggestions, we have made the following modifications to enhance the rigor of the analysis: We have added a multiple collinearity analysis among the features (Table 2), using the variance inflation factor (VIF) for evaluation. The results show that weekday (VIF = 7.07 > 5), indicating the presence of multiple collinearity, so this variable can be excluded. The absolute values of the correlation coefficients for holiday, rainfall, and wind speed are 0.1, 0.053, and 0.0088 respectively, and the correlations are relatively weak, so these variables can also be excluded.

Table 2. Variance Inflation Factor (VIF) Analysis for Features

Feature	VIF
Temperature	2.203
WindSpeed	1.864
Rainfall	1.805
Hour	4.145
Weekday	7.074
Weekend	3.668
Holiday	1.441

5.The attention mechanism introduces parameters U and b, but their dimensions and roles are not clearly explained. Clarifying whether these are shared across time steps or stations, and indicating tensor shapes, would improve the mathematical transparency.

Response: We would like to express our gratitude to the reviewers for their valuable suggestions. Based on the reviewers' recommendations, we have provided detailed explanations of the dimensions and functions of parameters U and b in formula (5). Specifically, $U \in \mathbb{R}^{d' \times d}$ is a learnable linear transformation matrix that projects the input features from h_t dimension d to a new feature space dimension d' (where d' is the potential dimension selected by the attention mechanism), while $b \in \mathbb{R}^{d'}$ is a learned attention vector used to weight the features for each time

step. U and b are shared across different time steps and different sites, enabling the model to make adaptive adjustments to temporal and spatial variations. We hope this will clarify the details in the equations and improve the clarity of the expression.

6.The STAGCN output is processed in parallel by LSTM and Informer models and then merged via an MLP. It is unclear why this architecture is favored over sequential stacking (e.g., LSTM → Informer). An ablation study exploring different fusion strategies could better justify the architectural design.

Response: In response to your comments, we have made the following revisions:

In the ablation experiment section, we have added a comparison with the sequential stacking architecture (LSTM → Informer). This is shown in Table 3 of the ablation experiment performance evaluation. The parallel fusion architecture outperforms the sequential stacking architecture and other baseline models in RMSE (4.036), MSE (2.692), and R2 (0.889). The output of STAGCN is processed in parallel by the LSTM and Informer models, and the features of different models are fused through a fully connected layer. This fusion method enables the model to simultaneously utilize the global information extraction ability of Informer and the local temporal relationship modeling ability of LSTM, which can better capture patterns at different time scales, improve the accuracy and robustness of the prediction, while the sequential stacking architecture may suffer performance degradation due to information compression.

7.There is a discrepancy between the abstract (RMSE = 4.036, MAE = 2.692) and Table 2 (RMSE = 4.021, MAE = 2.531). Please reconcile these numbers and ensure consistency throughout the manuscript.

Response: We are extremely sorry for our carelessness. Thank you for your reminder.

In the revised manuscript we submitted, we have unified the RMSE and MAE in the abstract and Table 2.

8.Although the Informer component is touted for its computational efficiency, no empirical evidence is provided. Including a table comparing training and inference times (e.g., FLOPs, seconds per epoch, GPU hours) across models would substantiate these claims.

Response: We fully agree with this suggestion and have made corresponding revisions to the manuscript.

We have added Table 4, which compares the differences in training among various models. The results show that compared with models like Transformer, the Informer model can shorten the training period. Moreover, thanks to its ProbSparse Attention mechanism, the complexity of the Informer model has been optimized from $O(L^2)$ to O(LlogL), verifying the computational efficiency advantage of the Informer model.

Table 4. Comparison of Model Calculation Efficiency

Model	FLOPs(M)	Training Time(s/epoch)
Informer	310.284	5.23
STAGCN	532.461	9.11
Transformer	480568	6.87
LSTM-Informer	430.251	9.89
STAGCN-LSTM-Informer	556.195	11.41

9.The use of only 61 days of data for evaluation raises concerns about model generalization. Testing the model on a temporally disjoint holdout set (e.g., a later month) would better assess seasonality effects and the robustness of the predictions.

Response: Thank you for raising concerns about the generalization ability of the model. We have made the following modifications:

1. To verify the generalization ability and prediction robustness of the model, we introduced an independent test set (in July 2021, consisting of 1,958,732 order data) in the experimental part to evaluate the model's performance in future time periods.

2. We added Table 5 and Fig 10 experiments to show the model's performance on the independent data set (the R^2 value on the original data set was 0.889, while the R^2 value on this data set was 0.872).

This confirms that the model has a strong adaptability to seasonal changes. However, we also acknowledge that future research should adopt more diverse data.

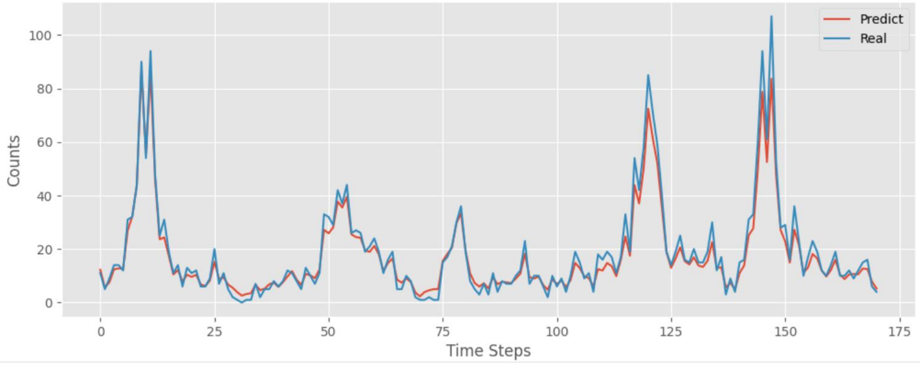

Fig 10. The prediction results of the independent test set

Table 5. Performance comparison on different test sets

Test Dataset	Model	RMSE	MAE	R^2
Original	STGACN-LSTM-Informer	4.036	2.692	0.889
Independent	STGACN-LSTM-Informer	4.215	2.813	0.872

Minor comments:

a) In table 1 headers. Instead of “300-meter buffer zone identification method” vs. “This paper identifies the method,” maybe go with “Baseline (300 m buffer)” and “Proposed.”

b) Table 2 title typo: “ST A GCN” —> “STAGCN.”

c) Section 4.1: remove the word “programmatically” from “Data acquisition was conducted programmatically via Python scripts…” (it’s redundant).

d) Figure 7’s y-axis needs units.

e) In the Related Work, Informer shouldn’t be in with “traditional” time-series methods, it is a recent Transformer variant.

Response: Thank you for your careful review. Based on your suggestions, we have thoroughly reviewed and revised the entire text, including chart titles, spelling errors of words, redundant sentences, and also regarding "d) Figure 7’s y-axis needs units.", in Fig 7, the y-axis represents the feature variables (classification labels), and it has no units. We have made every effort to ensure that each detail meets the requirements. Thank you for your patience and

guidance.
